# Optimization of tight gas reservoir fracturing parameters via gradient boosting regression modeling

**DOI:** 10.1016/j.heliyon.2024.e27015

**Published:** 2024-02-27

**Authors:** Huohai Yang, Xuanyu Liu, Xiangshu Chu, Binghong Xie, Ge Zhu, Hancheng Li, Jun Yang

**Affiliations:** a School of Petroleum Engineering, Southwest Petroleum University, Chengdu, China; b CNPC Greatwall Drilling Company, Beijing, China; c Sinopec Shengli Oilfield Hekou Oil Production Plant, Dongying, China; d Shunan Gas Field, PetroChina Southwest Oil and Gas Field Company, Luzhou, China; e Exploration and Development Research Institute of PetroChina Changqing Oilfield Company, Xi'an, China; f National Engineering Laboratory for Exploration and Development of Low-Permeability Oil & Gas Fields, Xi'an, China

**Keywords:** Tight gas reservoir, Hydraulic fracture, Data mining, Productivity prediction, Parameter optimization

## Abstract

In China, the exploitation of most unconventional oil and gas reservoirs is dependent on hydraulic fracturing, which is a key method employed when developing tight gas formations. Numerous scholars and field engineers, both domestically and internationally, have conducted extensive numerical simulations and physical experiments to study crack propagation and predict post-fracturing productivity in hydraulic fracturing. Although some progress has been reported in this regard, it is difficult to accurately predict the well productivity using mechanistic models owing to the vertical multilayered development of tight gas reservoirs. In this study, vertical fractured wells in a block of Sulige gas field were examined. The block relied on hydraulic fracturing to produce tight gases. However, as development progressed, the available reservoir environment deteriorated, large differences emerged between wells after fracturing, and the fracturing results did not meet the expectations. In this study, geological, construction, and generation data for this block that had been collected since 2007 were analyzed. After applying multiple machine-learning methods to filter outliers and fill in missing values, k-means clustering, classification enhancement, extreme gradient enhancement, and LightGBM algorithms were used to establish a regression model. The analysis results revealed that the regression accuracy of the cluster test set was as high as 70% and that the LightGBM model had the best regression effect among the 227 stripper wells in the block. After optimizing the fracturing construction parameters (fracturing fluid volume, proppant volume, liquid-nitrogen volume, and pumping rate), the average fracturing fluid and liquid-nitrogen volumes per well decreased, whereas the unit reservoir proppant and liquid-nitrogen volumes increased. The results also revealed that 182 wells showed an improved initial production capacity during fracturing. The average gas production index per meter increased by 22.04%. This approach enabled rapid and efficient production forecasting and construction optimization. Moreover, this represents a novel fracture design method that is applicable to onsite engineers in tight gas production fields in the Ordos region.

## 1 Introduction

In the oil and gas production process, rapid and accurate prediction of preconstruction productivity is critical in the oilfield department, and subsequent measures should be taken to enable stripper wells to achieve enhanced oil recovery. With the accumulation of relevant data and advancements in machine learning in recent decades, the applications of machine learning in oil and gas production have increased. Although several relevant studies have been conducted recently, owing to the shortage of algorithms and uncertainty in the oil and gas industry, a complete theoretical system and sample database have not yet been developed in the field of oil and gas intelligence [1]. Selecting a reasonable method to analyze the geological conditions and construction method of a block is critical for ensuring effective analysis.

Currently, there are two main approaches for optimizing the design of hydraulic fracturing parameters: crack inversion and production simulation methods. The crack inversion method primarily focuses on fracturing parameters, reservoir characteristics, and economic factors. By simulating and analyzing the expansion of hydraulic fractures, the formed fractures could be described, enabling the establishment of a matching relationship between the fracturing fractures and reservoir properties. This provides guidance and suggestions for optimizing fracturing parameters. Jiang et al. [2] proposed a new method that combines DPVS model, reservoir numerical simulation, and reservoir classification to optimize fracture parameters of heterogeneous horizontal gas wells. Jiang et al. [3] provided theoretical support for horizontal wells in the BZ oilfield, establishing physical fracturing models and productivity prediction mathematical models. Specifically, they focused on the impact of fracture parameters and injection wells on comprehensive production. Salah et al. [4] improved the production of horizontal multi-stage fracturing wells, reduced costs, and increased profits by integrating rock physics, geomechanics, and production data. However, the effectiveness of parameter optimization is significantly dependent on the accuracy and reliability of the hydraulic fracture model. Additionally, hydraulic fracture simulations are computationally intensive, and performing full-wellbore crack-inversion simulations along the horizontal section of a well is time-consuming and labor-intensive.

The production simulation method utilizes numerical reservoir simulation techniques. Based on the accuracy of fitting historical production data with numerical models, different fracture parameter schemes were established and executed. Specifically, production was considered a constraint for parameter optimization. Yu et al. [5] used response surface methodology, combined with hydraulic fracturing numerical simulation and economic analysis, to maximize NPV and optimize the production efficiency of unconventional gas reservoirs by considering key parameters. Rammy et al. [6] optimized the hydraulic fracturing parameters and horizontal well length of shale gas reservoirs via differential evolution to improve economic efficiency. Li et al. [7] significantly improved the gas production of coalbed methane wells via optimization of construction parameters. Xu et al. [8] revealed the relationship between hydraulic fracturing parameters under different geological conditions and uniform crack propagation, SRV, and NPV. This production simulation method offers better real-time performance and flexibility. It allows the visualization and presentation of results, facilitating understanding and analysis. However, it has higher input data requirements, and significant computational resources and time are required when dealing with large-scale and complex reservoir systems.

With the advancement of computer performance and urgent need for data processing, data mining methods, such as machine learning and deep learning, have provided new approaches for optimizing fracturing parameters. Many scholars are currently attempting to leverage these methods, breaking through previous assumptions and limitations and using data-driven models in conjunction with field production data for fracturing parameter optimization. Researchers, such as Koroteev et al. [9], analyzed the application of artificial intelligence in the upstream field of oil and gas, emphasizing risk reduction, process acceleration, and data, personnel, and collaboration challenges. Sircar et al. [10] reviewed the latest advances in machine learning and artificial intelligence technologies in data processing and interpretation to improve performance as well as reduce risks and costs. Aung et al. [11] summarized the applications of artificial neural networks and support vector machines in geological data interpretation, price prediction, and flow regime prediction to improve exploration and production efficiency. Choubey et al. [12] reviewed the applications of artificial intelligence and machine learning technologies in the oil and gas industry, spanning from exploration to distribution. They highlighted their crucial role in big data utilization and decision-making. Wang et al. [13] and Zhou et al. [14] established data mining models to understand the relationship between parameters and production capacity and optimize fracturing parameters based on the optimization model. Moreover, other studies by Al Mudhafar [15] and Pankaj et al. [16] utilized surrogate models to optimize fracturing parameters.

Based on local and global research, data mining technology has been shown to be applicable in the field of oil and gas development. Its applications mainly include reservoir parameter prediction, fracturing effect prediction, well and layer selection for fracturing, and fracturing decision-making. However, there remain some challenges in the application of data mining techniques to optimize fracturing processes in oil and gas field development.

First, predictive models based on data mining methods primarily focus on production as the target variable; however, production varies significantly under different production systems.

Second, multiple factors influence the effectiveness of fracturing, and previous studies focused on different factors. Some researchers considered fewer factors, leading to a less comprehensive analysis or an unequal treatment of different factors.

Fracturing is the primary stimulation method, and the production after fracturing is one of the main indicators for evaluating the fracturing effect. Establishing a prediction model for production after fracturing and optimizing the fracturing parameters, based on the prediction results, can improve production and increase natural gas recovery. However, owing to the harsh construction environment, it is difficult to record data, and thereby, data loss often occurs, causing difficulties in forecasting production after fracturing. Therefore, after consulting with field engineers, we addressed the issue of missing critical fracturing parameters in the field. Outliers were removed, and data interpolation models using random forest, KNN, and miceforest [17] were developed to populate the missing data. The optimal dataset was then chosen for production capacity prediction. If prediction accuracy was insufficient, then we implemented a clustering process prior to prediction and subsequently selected the best prediction model for each production level. For the stripper wells, an optimization model grounded in a genetic algorithm was created, and a regression model was used to determine the relationship between each influencing factor and the meter recovery index. After evaluating various models, the LightGBM stripper well productivity prediction model was chosen as the most accurate. An optimization model was then crafted using a genetic algorithm, focusing on refining four factors: the quantities of fracturing fluid, proppant, liquid nitrogen, and construction displacement. This resulted in an optimal 22.04% increase in gas production. Additionally, it reduced fracturing costs and enhanced the efficiency of fracturing agents. When compared to the conventional numerical simulation methods in the industry, our proposed approach exhibits greater accuracy and speed, aiding in identifying and addressing field challenges. Theoretically, it augments the output of stripper wells while bolstering fracturing efficiency and economic benefits.

## 2 Geological overview of the target block

**Fig. 1:**
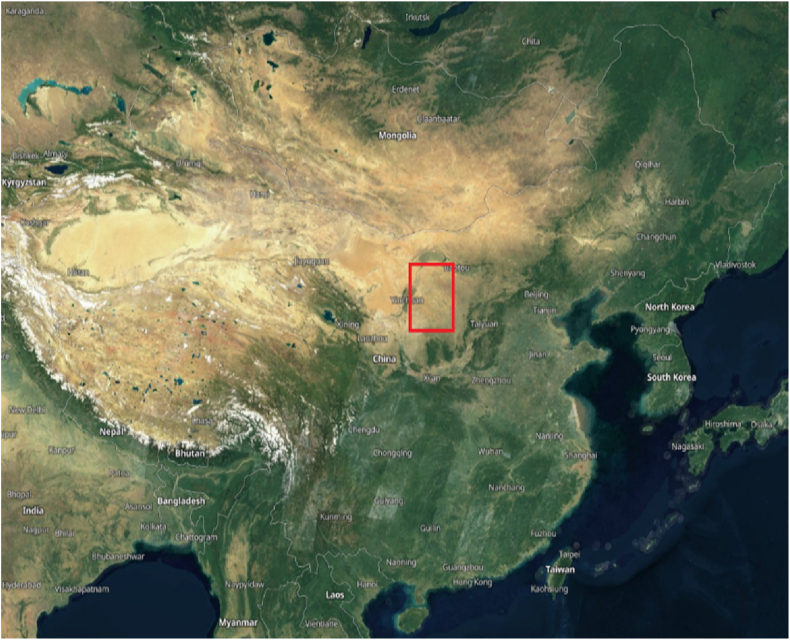
Location of the research area (from Google Maps).

The operation block is located in the northern part of the Sulige gas field (Fig. 1), with an area of 1162 km^2^ and a natural gas geological reserve of 177.716 billion m^3^. Since the fracturing operation began in 2007, the size of the enriched area has decreased annually with the development of interlayers. The thin-interlayer hydraulic fracture characteristics are affected by the interlayer, which is difficult to extend, and it is difficult to ensure that the fractures extend through the reservoir and are linked to an effective sand body. Currently, the focus is on the sub-enrichment regions of natural gas and extraction of thin interbedded reservoirs. Due to the considerable variability in construction parameters during the fracturing process, issues, such as proppant plugging and thin interbedded channeling, can arise. These issues can lead to a less effective fracturing stimulation than anticipated, thus impacting the block's recovery efficiency. Concurrently, the porosity and permeability of the succession area have diminished, and the fracturing fluid poses potential damage to the reservoir. With low gas saturation and inadequate natural energy, these characteristics are indicative of a low-gas reservoir [[18], [19], [20]].

## 3 Data processing and characterization engineering

### 3.1 Data processing

**Fig. 2:**
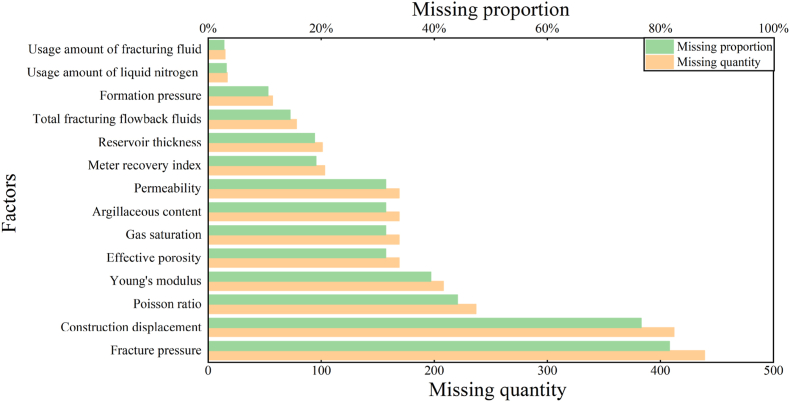
Missing dataset for the Su A area.

Prior to data analysis, block reservoir and production data were collected and processed. The selection of evaluation indicators corresponds to the first step in developing a fracturing database that establishes prediction models based on evaluation indicators. The raw data used in this study were obtained from the production construction system database of the fracturing unit in the Su A Block. The acquired raw data were sorted, and factors related to the production capacity were screened and classified into reservoir physical, rock mechanics, and fracturing construction parameters. These classifications pertain to 538 production wells. The details are presented in Table 1 and Fig. 2.

**Table 1:** Factors affecting the productivity of the vertical wells.

Reservoir physical parameters	Rock mechanics parameters	Construction fracturing parameters
Formation pressure	Poisson's ratio	Usage amount of fracturing fluid
Effective porosity	Young's modulus	Usage amount of proppant
Gas saturation	Fracture pressure	Usage amount of liquid nitrogen
Argillaceous content		Total fracturing flowback fluids
Permeability		Construction displacement
Reservoir thickness

Production is primarily influenced by reservoir geology and post-fracturing construction. Throughout the production process, the factors impacting productivity are multifaceted and dynamic. Generally, the open flow rate and daily gas production rate can indicate the production status of a gas well. However, in the target block, most gas wells are not evaluated during this process. Based on a summary and analysis of production experiences, the wellhead casing pressure was integrated with cumulative gas production. This combination was then used to define the capacity index based on the output per unit of production pressure difference (Eq. (1)).

(1) $$J=\frac{Q_{f}}{h\Delta p_{f}}$$

Based on this, the productivity derived from the meter recovery index was substituted with the average pressure drop yield observed 90 days post-fracturing construction, serving as a representation of the post-pressure production capacity. Specifically, the daily average pressure drop in gas production, per unit thickness of the reservoir within the 90 days following construction, was employed as the meter recovery index. The calculation is as shown in Eq. (2).

(2) $$J=\frac{\sum_{i=1}^{90}\frac{q_{g}}{p_{cf1}-p_{cf2}}}{h\cdot 90}$$

### 3.2 Characterization engineering

**Fig. 3:**
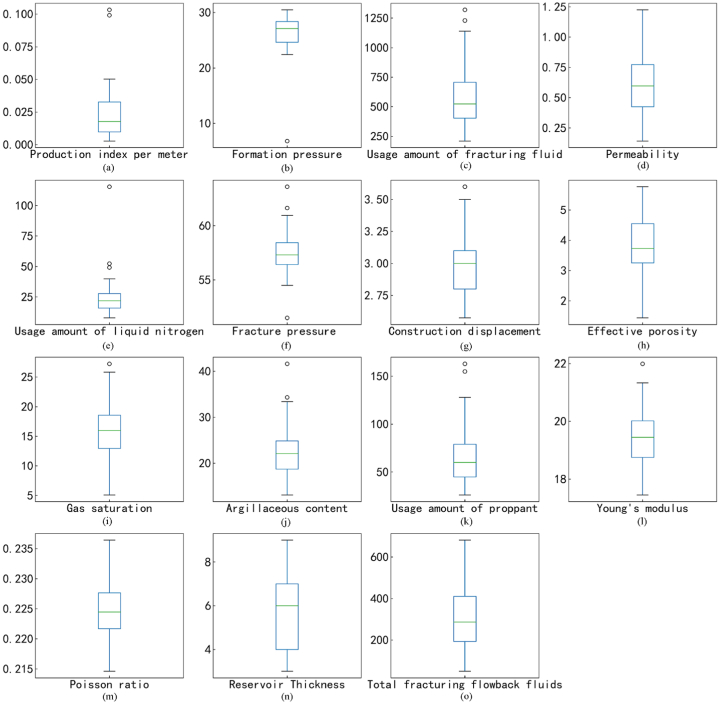
Outlier recognition (2020 as an example).

Due to the limitations of the field equipment, a significant amount of data was missing in the collected set. Typically, the challenge of missing data is tackled by filling features with a low rate of missing data using their mean value and discarding features with a high missing data percentage and no discernible pattern. However, these types of methods can distort the original data distribution and diminish valuable insights. A more effective approach involves maximizing rational data interpolation. In this research, outlier detection was employed (as shown in Fig. 3 (a)–(o)) alongside random forest and KNN techniques, to identify outliers and utilize multiple interpolation strategies to populate the missing values. The random forest method is adept at managing high-dimensional data and remains accurate even when many features are missing. KNN is less sensitive to outliers, and it offers high accuracy in filling data. Interpolation techniques can be single or multiple in nature. Although single interpolation is straightforward, it often falls short in addressing data uncertainty. Multiple interpolations, on the other hand, can mitigate these shortcomings through various functions and models. Given the lack of a well-established theoretical foundation for devising a construction plan in the field, and the high variability among construction parameters, this study employed three distinct methods to address missing values. Their effects were then compared. Following a final selection and reduction process, 444 data entries were preserved for further analysis. The outcomes of the data-filling procedures of the three methods are detailed in Table 2.

**Table 2:** Results obtained with different data-filling methods.

Factor	Random forest		KNN		Miceforest	
	Variance	Standard error	Variance	Standard error	Variance	Standard error
Usage amount of fracturing fluid	67,460.51	12.33	69,561.65	12.52	65,885.92	12.17
Usage amount of proppant	773.64	1.32	807.66	1.35	781.04	1.33
Usage amount of liquid nitrogen	63.51	0.38	66.40	0.39	63.21	0.37
Total fracturing flowback fluids	12,721.52	5.38	13,324.03	5.48	14,168.92	5.63
Formation pressure	4.82	0.10	5.76	0.11	4.93	0.11
Effective porosity	0.73	0.04	0.86	0.04	0.64	0.03
Gas saturation	29.78	0.27	37.28	0.29	29.58	0.26
Argillaceous content	39.21	0.32	28.65	0.25	23.97	0.23
Permeability	0.07	0.01	0.07	0.01	0.06	0.01
Reservoir thickness	8.95	0.14	10.76	0.16	8.83	0.14
Poisson ratio	0	0	0	0	0	0
Young's modulus	2.28	0.07	1.74	0.06	1.13	0.05
Fracture pressure	0.91	0.05	5.60	0.11	1.43	0.06
Construction displacement	0.01	0.01	0.05	0.01	0.02	0.01
SUM	81,105.94	20.43	83,850.51	20.78	80,969.68	20.40

**Fig. 4:**
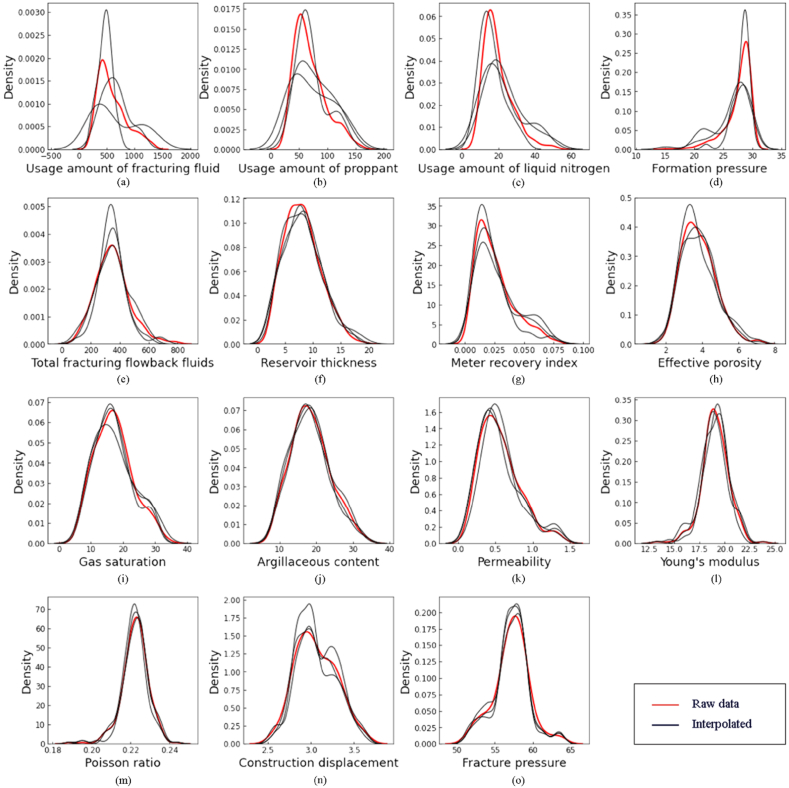
Miceforest interpolation effect.

By comparing the filling errors of the different algorithms listed in Table 2, the results of the multiple interpolations are selected as samples for subsequent research. The effects of filling in the data are shown in Fig. 4 (a)–(o).

For processed data, the difference between variables should be maximally preserved, whereas the influence of different orders of magnitude should be eliminated. When the processed data are combined with the field demand, the higher the production efficiency, the better the production effect. Therefore, when the data have *m* features and each feature has *n* samples, Eq. (3) can be selected to process the data and eliminate dimensional effects.

(3) $$Y_{i}\left(k\right)=\frac{x_{i}\left(k\right)-\min\;x_{i}\left(k\right)}{\max\;x_{i}\left(k\right)-\min\;x_{i}\left(k\right)},i\in \left(1,2,\ldots \ldots ,m\right),k\in \left(1,2,\ldots \ldots ,n\right)$$

## 4 Data cluster and productivity prediction

### 4.1 Productivity regression model

**Fig. 5:**
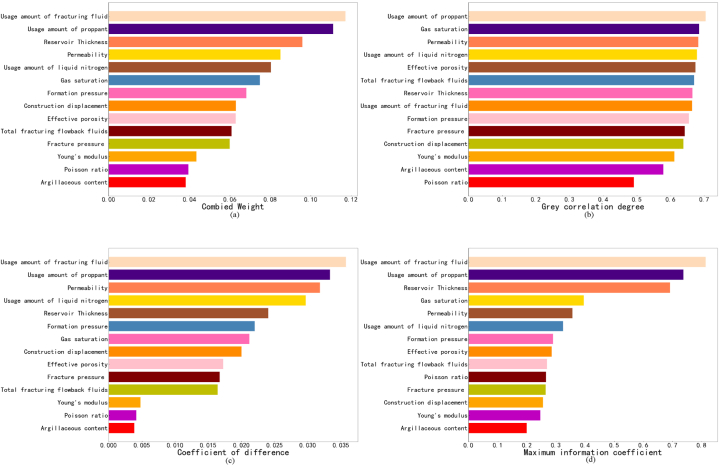
Weight calculation results obtained with different models.

Evaluating productivity is of great significance for oilfield development and production. It can be used to evaluate and improve the preliminary exploration results and provide a reference for the design of construction methods. After data pre-processing, we introduced a weight analysis step. We can understand the degree of contribution of different factors to productivity and further optimize the model via a weight analysis. In this process, the grey correlation, entropy weight, and maximum information coefficient were selected to consolidate the results and avoid the influence of a single model contingency. The correlation results are presented in Fig. 5 (a)–(d) and Table 3.

**Table 3:** Weight values and rankings of each method.

Feature name	Combined weight		Grey correlation		Entropy weight method		Maximum information coefficient	
	sort	weight	sort	weight	sort	weight	sort	weight
Usage amount of fracturing fluid	1	0.1172	8	0.0735	1	0.1272	1	0.1508
Usage amount of proppant	2	0.1111	1	0.0779	2	0.1187	2	0.1366
Reservoir thickness	3	0.0958	7	0.0736	5	0.0856	3	0.1281
Permeability	4	0.0850	3	0.0755	3	0.1132	5	0.0661
Usage amount of liquid nitrogen	5	0.0803	4	0.0751	4	0.1056	6	0.0601
Gas saturation	6	0.0749	2	0.0758	7	0.0754	4	0.0734
Formation pressure	7	0.0682	9	0.0724	6	0.0783	7	0.0538
Construction displacement	8	0.0630	11	0.0706	8	0.0712	12	0.0474
Effective porosity	9	0.0629	5	0.0745	9	0.0614	8	0.0529
Total fracturing flowback fluids	10	0.0608	6	0.0741	11	0.0584	9	0.0500
Fracture pressure	11	0.0599	10	0.0710	10	0.0595	11	0.0491
Young's modulus	12	0.0434	12	0.0677	12	0.0170	13	0.0457
Poisson ratio	13	0.0395	14	0.0543	13	0.0148	10	0.0492
Argillaceous content	14	0.0382	13	0.0640	14	0.0137	14	0.0368

**Fig. 6:**
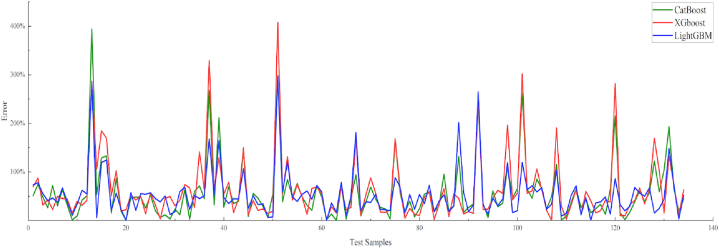
CatBoost, XGBoost, and LightGBM model regression error comparison.

After studying and comparing various methods, CatBoost [[21], [22], [23]], XGBoost [[24], [25], [26], [27]], and LightGBM [[28], [29], [30], [31]] were selected, and the results of the weighted summation were weighted to establish a model for predicting the gas meter recovery index. In this study, 30% of the data were randomly selected as the test set. The model results are presented in Table 4 and Fig. 6.

**Table 4:** CatBoost, XGBoost, and LightGBM model regression error statistics.

Regression model	Prediction error
CatBoost	58.24%
XGBoost	63.51%
LightGBM	54.84%

As shown in Table 4, the LightGBM exhibited the best regression effect; however, the regression errors of the three models remained unacceptable for field applications. To solve this problem, a clustering algorithm was used to further process the sample dataset. Given the influence of several factors on reservoir productivity, it is difficult to obtain quantitative predictions. The classification of the productivity levels of the producing wells not only constrains and guides quantitative predictions but also improves the accuracy of the quantitative prediction based on the classification.

### 4.2 Data cluster

Cluster analysis is an unsupervised machine learning algorithm and is an important technique for mining data distributions and hidden patterns [32]. Based on the principle of minimizing the distance within a group and maximizing the distance outside the group, samples can be grouped according to data similarity without a given classification. The algorithm uses the Euclidean distance to measure the distance from the sample to the cluster center and uses the error sum of squares, *SSE*, as an objective function to measure the effect of clustering. The classification result with the smallest *SSE* is selected as the final result.

The Euclidean distance formula is as shown in Eq. (4).

(4) $$dist\left(i,j\right)=\sqrt{{\left(x_{i1}-x_{j1}\right)}^{2}+{\left(x_{i2}-x_{j2}\right)}^{2}+\ldots \ldots +{\left(x_{in}-x_{jn}\right)}^{2}}$$

where characteristic $x_{i}=\left(x_{i1},x_{i2},\ldots x_{in}\right)$, and characteristic $x_{j}=\left(x_{j1},x_{j2},\ldots x_{jn}\right)$.

(5) $$SSE={\sum_{xeE_{1}}dist\left(e_{1},x\right)}^{2}+{\sum_{xeE_{2}}dist\left(e_{2},x\right)}^{2}+\ldots \ldots +{\sum_{xeE_{n}}dist\left(e_{n},x\right)}^{2}$$

In Eq. (5), *E*_*i*_ denotes the ith cluster, *e*_*i*_ denotes the center of the ith cluster and *x* denotes the sample data of the cluster.

**Fig. 7:**
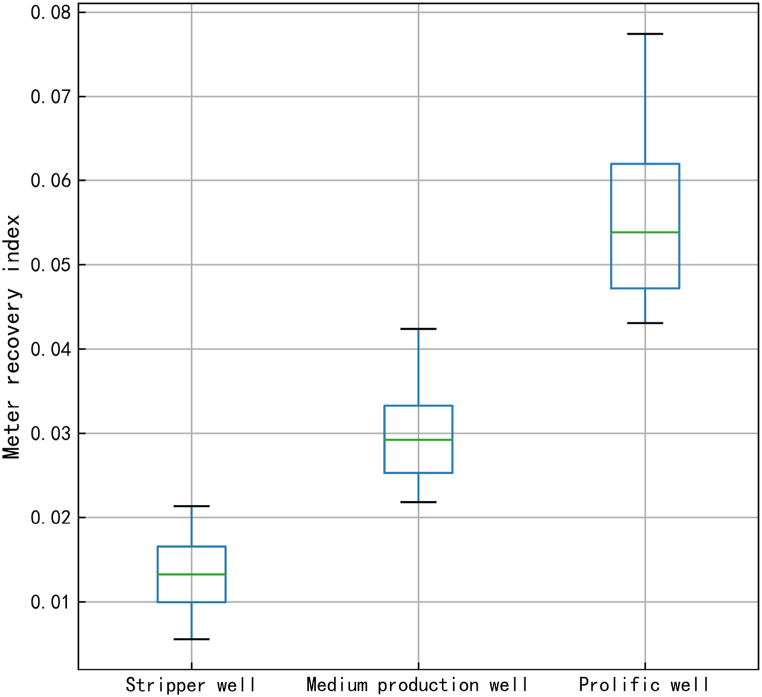
Clustered boxplot of the metered gas production index.

The optimal number of categories was determined to be three by employing three to five clustering centers on the samples and conducting iterative calculations using the silhouette coefficient to compare and analyze clustering effectiveness. Furthermore, the classification ranges of stripper, middle production, and prolific wells were determined based on the classification results (Table 5). The yield distributions of the wells are shown in Fig. 7.

**Table 5:** Cluster well meter gas recovery index distribution.

	Stripper well	Middle production well	Prolific well
Classification range (10^4^ m^3^/MPa·m)	<0.0215	0.0215–0.0425	>0.0425
Average (10^4^ m^3^/MPa·m)	0.0133	0.0299	0.0554
Median (10^4^ m^3^/MPa·m)	0.0132	0.0292	0.0539
Upper quarter (10^4^ m^3^/MPa·m)	0.0165	0.0333	0.0620
Lower quarter (10^4^ m^3^/MPa·m)	0.0099	0.0252	0.0472
Upper bound (10^4^ m^3^/MPa·m)	0.0213	0.0424	0.0774
Lower bound (10^4^ m^3^/MPa·m)	0.0055	0.0218	0.0430

### 4.3 Regression of wells with different productivities

**Fig. 8:**
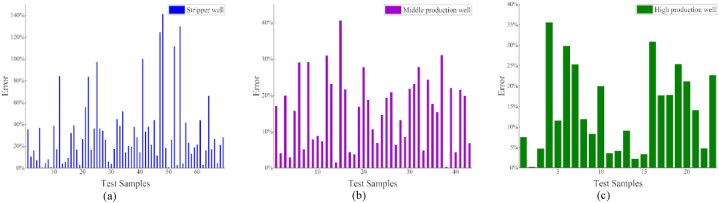
Regression effect of XGBoost model after clustering.

After grouping the data using a clustering algorithm, a regression model was applied. Compared to the results before clustering (Table 3), the results obtained after clustering demonstrate that the regression model significantly improves data accuracy. Furthermore, the model accuracy is substantially improved when compared to that of the previous overall regression by counting the data after the classification regression, as shown in Fig. 8 (a)–(c) to Fig. 10 (a)–(c).

**Fig. 9:**
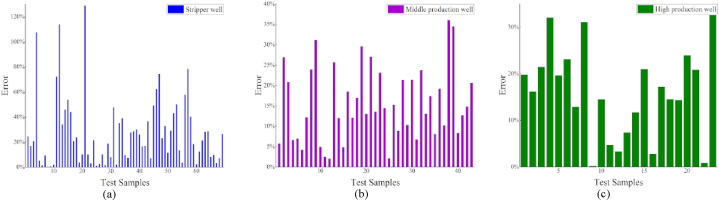
Regression effect of LightGBM model after clustering.

**Fig. 10:**
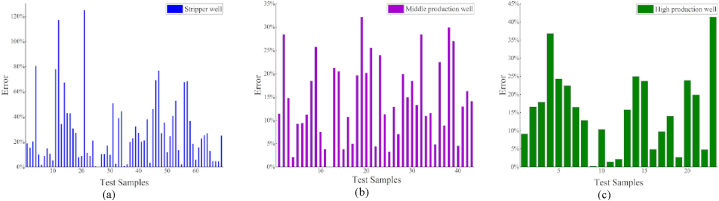
Regression effect of CatBoost model after clustering.

The best models for the various well types are determined by comparing the errors of the different models, as listed in Table 6. The regression results of the three models were similar for the middle and prolific production wells. Moreover, the LightGBM regression yielded the most favorable results for the stripper wells, achieving an *R*^2^ value of 0.66.

**Table 6:** Comparison of the model error statistics after clustering.

	Stripper well	Middle production well	Prolific well
Tested sample amount	69	43	23
Average single-well prediction error (CatBoost)	32.3%	15.8%	14.4%
Average single-well prediction error (XGBoost)	27.8%	14.5%	15.5%
Average single-well prediction error (LightGBM)	27.2%	15.4%	15.9%
Average error	29.1%	15.2%	15.3%

## 5 Optimization of the fracturing parameters for low-yield wells

### 5.1 Algorithm principle

**Fig. 11:**
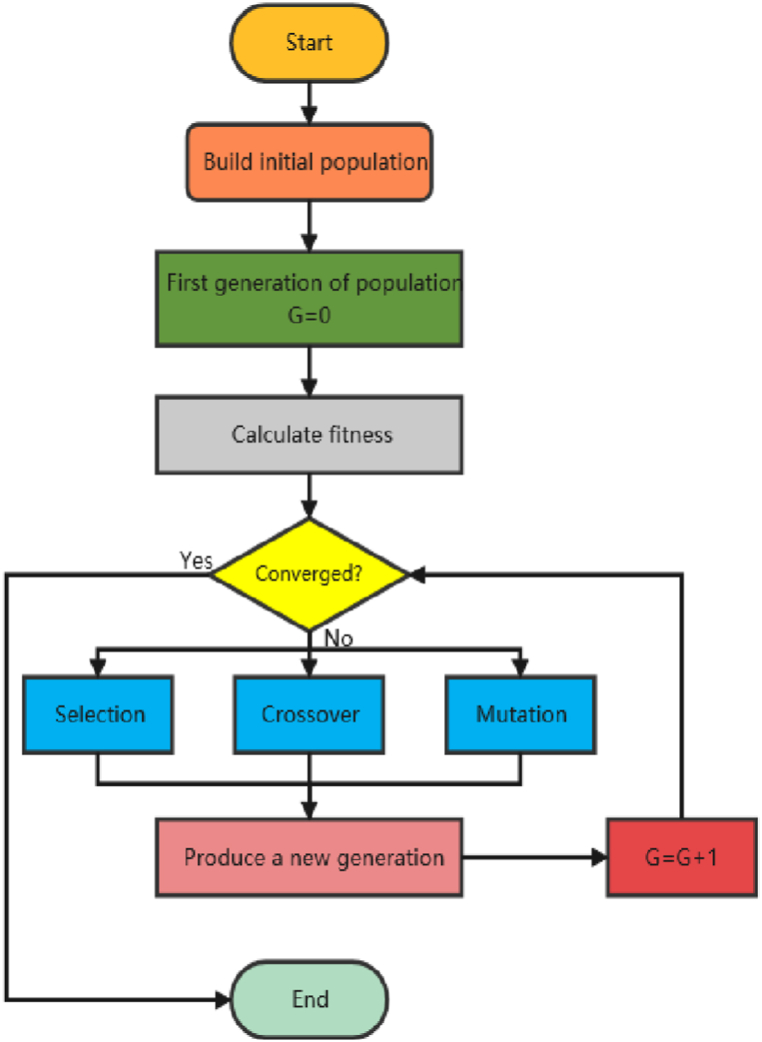
Flowchart of the genetic algorithm.

According to the results of previous studies, it is nearly impossible to solve the problem directly based on the exploration of oil and gas combined with the field production demand. Therefore, a machine learning model was introduced for engineering design optimization and genetic algorithm was used to solve it. Considering the highest gas production index per meter as the goal, the LightGBM regression model with the best performance in the regression analysis of the stripper wells was used as the objective function to establish an optimization model. A flowchart of the algorithm is shown in Fig. 11. The algorithm continuously modifies the initial pseudorandom population to reach a local minimum position. Initially, each member of the pseudo-random population is a potential solution to the problem. Subsequently, following a few iterations, the genetic algorithm guides the population to the best-fit position [[33], [34], [35], [36]]. Under fixed values of the physical parameters of the reservoir and mechanical parameters of the rock, the maximum number of iterations was set to 100, the initial population number was 50, and the fracturing parameters were optimized with the boundary value of the fracturing parameters as the constraint condition. Thus, the optimization model can be expressed as shown in Eq. (6). Given that the model does not provide a complete objective function, it learns the LightGBM regression model of low-producing wells during optimization, obtains it as the objective function, and sets the maximum value of the construction parameter column as the constraint condition for the next analysis.

(6) $$\begin{array}{c} \left(MP\right)\;\max\;f\left(X\right)\\ s.t.\;\min\;C_{i}\left(X_{j}\right)\leq C_{i}\left(X\right)\leq \max\;C_{i}\left(X_{j}\right)\;\left(i=1,2,3,4;\;j=1,2,\ldots \ldots ，227\right) \end{array}$$

### 5.2 Fracturing parameter optimization application

**Fig. 12:**
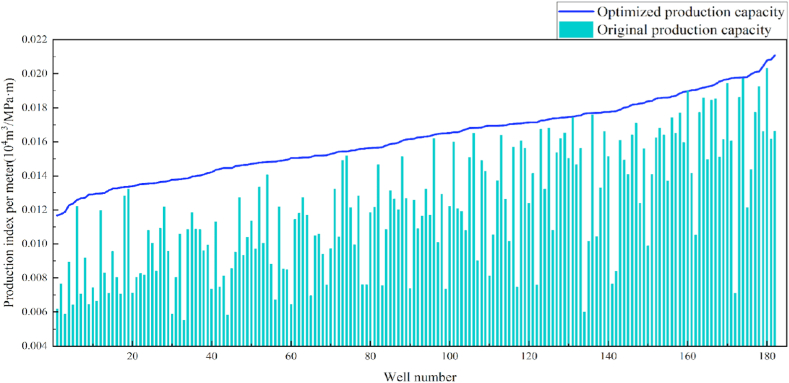
Comparison of productivity before and after optimization.

An optimization model based on a genetic algorithm and LightGBM low-producing well regression model was established to optimize 227 stripper well samples. The optimization results demonstrate that all the wells can be optimally fitted before the maximum number of iterations. A total of 182 production wells were optimized. The improvements obtained are listed in Table 7 and Fig. 12.

**Table 7:** Optimization model effect statistics.

Improvement effect of meter recovery index	Well quantity
<20%	59
20–40%	51
40–60%	19
60–80%	21
80–100%	12
>100%	20

**Fig. 13:**
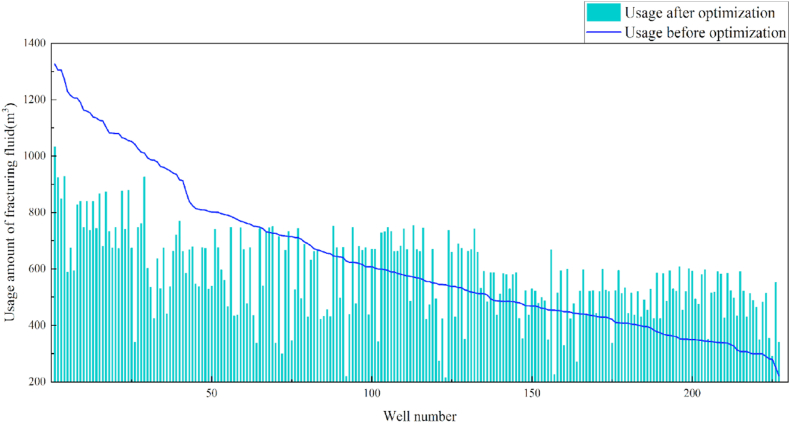
Comparison of the fracturing fluid usage before and after optimization.

**Fig. 14:**
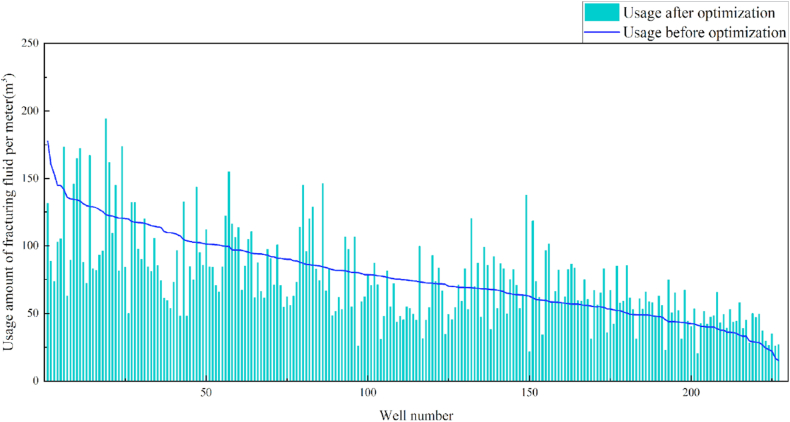
Comparison of the fracturing fluid usage before and after optimization (unit reservoir).

**Fig. 15:**
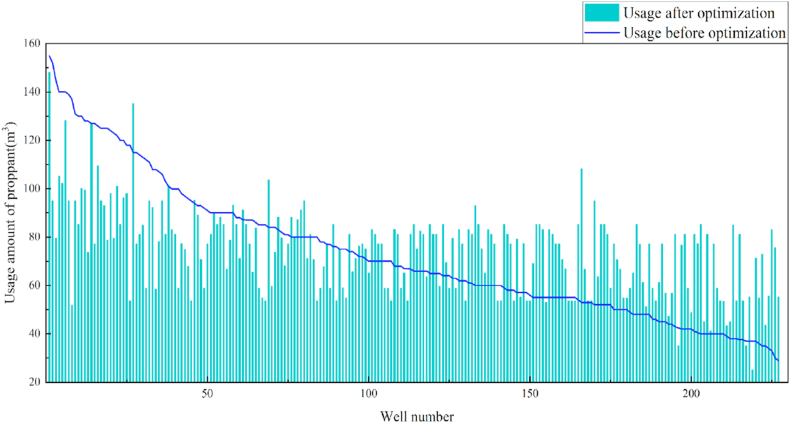
Comparison of the proppant usage before and after optimization.

**Fig. 16:**
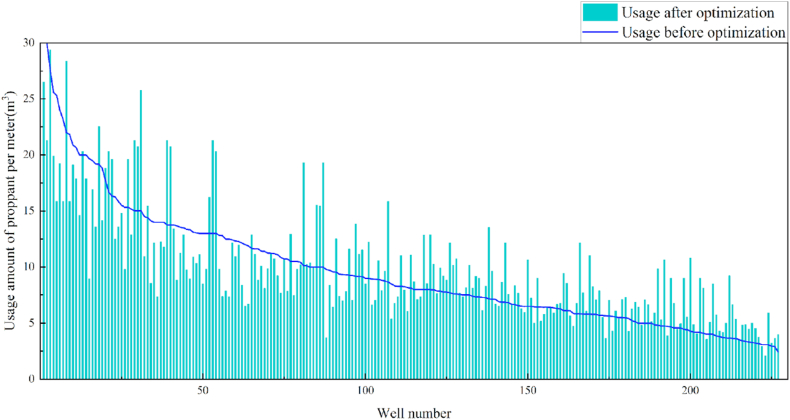
Comparison of the proppant usage before and after optimization (unit reservoir).

**Fig. 17:**
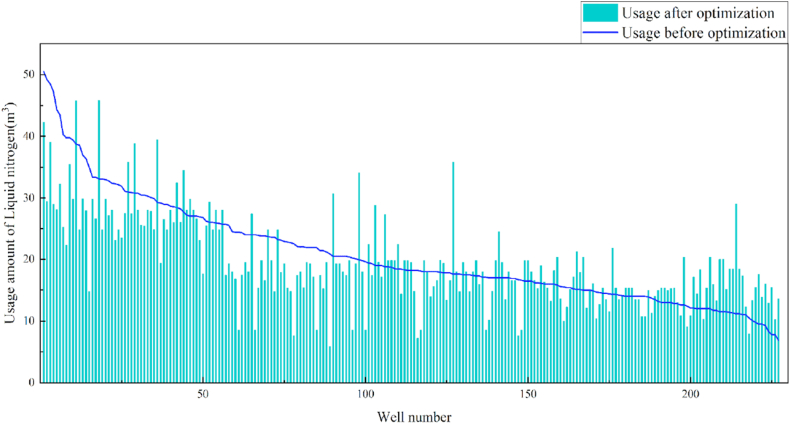
Comparison of the liquid nitrogen usage before and after optimization.

**Fig. 18:**
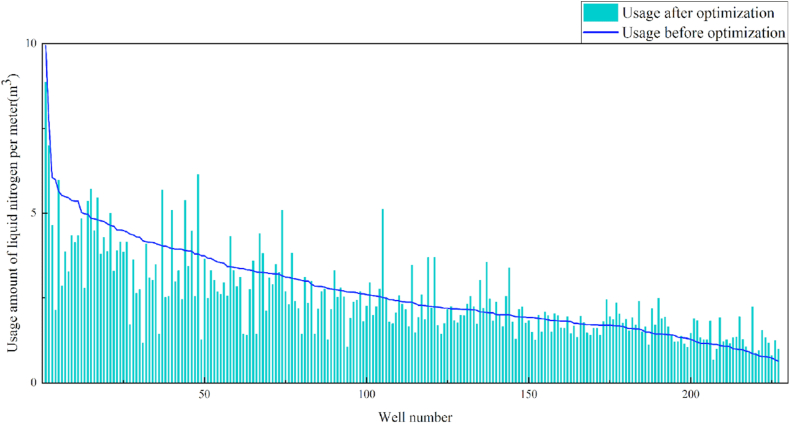
Comparison of the liquid nitrogen usage before and after optimization (unit reservoir).

**Fig. 19:**
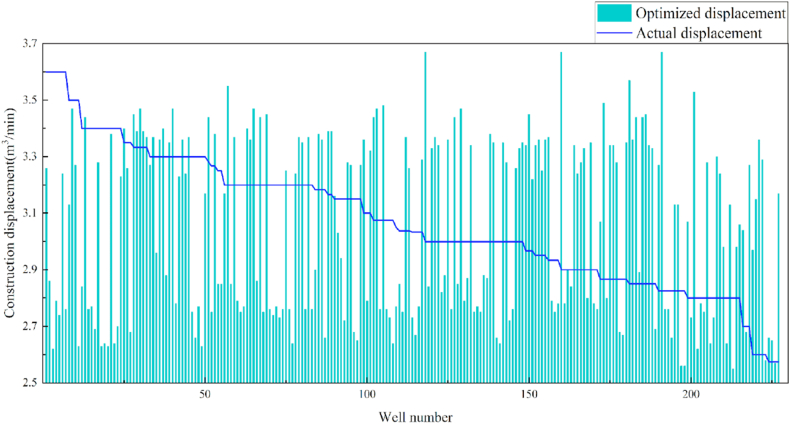
Comparison of the construction displacement before and after optimization.

In terms of the construction parameters, the overall proppant, flowback fluid volume, and construction displacement did not change significantly before or after model optimization. However, the average fracture fluid and liquid nitrogen usage per well reduced by 8.6% and 6.3%, respectively, which can be used as a reference for field cost control. Given that many uncontrollable factors affect flowback, formation water was added when calculating the amount of flowback liquid, which resulted in a serious discrepancy between the statistical and actual results; therefore, the model was not suitable for flowback optimization. Additionally, vertical wells involve reservoir thicknesses ranging from 3 to 19 m. Therefore, the optimization results were further translated into unit reservoir thickness changes to better reflect the optimized parameters. The results revealed that the proposed approach increased the proppant and liquid nitrogen usage by 7.3% and 7.1%, respectively, when compared with the current protocol. The changes in the construction parameters before and after optimization are shown in Table 8 and Fig. 13, Fig. 14, Fig. 15, Fig. 16, Fig. 17, Fig. 18, Fig. 19.

**Table 8:** Comparison of the average values of the construction parameters before and after optimization.

Construction parameters	Before optimization	After optimization	Before optimization (unit reservoir)	After optimization (unit reservoir)
Usage amount of fracturing fluid (m^3^)	629.69	575.45	77.62	74.67
Usage amount of proppant (m^3^)	73.19	74.21	9.05	9.71
Usage amount of liquid nitrogen (m^3^)	20.79	19.48	2.68	2.49
Total fracturing flowback fluids (m^3^)	372.62	369.73	47.16	49.41
Construction displacement (m^3^/min)	3.07	3.08	–	–

## 6 Conclusions

Several machine learning models have been used to process and analyze the missing oil and gas data. The main conclusions of this study are as follows.

(1) In the dataset analyzed, the multiple interpolation method outperforms the other techniques. It preserves the relationship between variables and accurately simulates the distribution of missing data. However, due to the constraints of the data source, certain important variables—identified from field experience—were excluded during statistical analysis because of their lack of diversity. In subsequent studies, there should be an emphasis on broadening the sample collection and enhancing the quality of the source data to ensure the study's adaptability.
(2) Theoretically, the block achieved an initial stimulation of 22.04% after killing the stripper wells. Reasonable fracturing design parameters can be developed according to the static parameters of each well, and the capacity can be predicted before construction. Hence, it is possible to adjust unreasonable development schemes in a timely manner. The aforementioned workflow can improve economic efficiency, while reducing risks in the field production process. Hence, its application is valuable.
(3) The prediction accuracy of the model for middle- and high-production wells was as high as 85%, and the prediction accuracy for stripper wells reached 70%, which could be further enhanced. However, the unavoidable presence of groundwater during on-site backflow analysis suggests that results could be further refined and optimized using methods such as enhanced analytical techniques.

## Symbol comment

J is the oil well productivity index (m³/(MPa·m)), $Q_{f}$ is the oil well production (t/d), $\Delta p_{f}$ is the oil well production differential pressure (MPa), $q_{g}$ is the daily gas production of the well (10^4^ m^3^/d), $p_{cf1}$ is the maximum casing pressure of the day (MPa), $p_{cf2}$ is the minimum casing pressure produced in the same day (MPa), h is the effective reservoir thickness (m), and C(X) indicates the construction parameters.

## CRediT authorship contribution statement

**Huohai Yang:** Conceptualization. **Xuanyu Liu:** Conceptualization. **Xiangshu Chu:** Writing – review & editing, Writing – original draft, Visualization, Methodology, Data curation, Conceptualization. **Binghong Xie:** Visualization. **Ge Zhu:** Data curation. **Hancheng Li:** Data curation. **Jun Yang:** Data curation.

## Declaration of competing interest

The authors declare no competing financial interests or personal relationships that may have influenced the results of this study.

## References

[1] Min C., Dai B., Zhang X. (2020). A review on application progress of machine learning in oil and gas industry. Journal of Southwest Petroleum University (Science & Technology Edition).

[2] Jiang B., Li H., Zhang Y. (2016). Multiple fracturing parameters optimization for horizontal gas well using a novel hybrid method. J. Nat. Gas Sci. Eng..

[3] Xu W.J., Hu Y.Q., Zhao J.Z. (2014).

[4] Salah M., Ibrahim M. (2018). SPE Annual Technical Conference and Exhibition.

[5] Yu W., Sepehrnoori K. (2013). Optimization of multiple hydraulically fractured horizontal wells in unconventional gas reservoirs. Journal of Petroleum Engineering.

[6] Rammay M.H., Awotunde A.A. (2016). Stochastic optimization of hydraulic fracture and horizontal well parameters in shale gas reservoirs. J. Nat. Gas Sci. Eng..

[7] Li S., Bian L., Huang M. (2020).

[8] Xu S., Guo J., Feng Q. (2022). Optimization of hydraulic fracturing treatment parameters to maximize economic benefit in tight oil. Fuel.

[9] Koroteev D., Tekic Z. (2020). Artificial intelligence in oil and gas upstream: trends, challenges, and s-cenarios for the future. Energy and AI.

[10] Sircar A., Yadav K., Rayavarapu K. (2021). Application of machine learning and artificial intelligence in oil and gas industry. Petroleum Research.

[11] Aung Z., Mikhaylov I.S., Aung Y.T. (2020). 2020 IEEE Conference of Russian Young Researchers in Electrical and Electronic Engi-Neering (EIConRus).

[12] Choubey S., Karmakar G.P. (2021). Artificial intelligence techniques and their application in oil and gas industry. Artif. Intell. Rev..

[13] Wang L., Yao Y., Wang K. (2022). Data-driven multi-objective optimization design method for shale gas fracturing parameters. J. Nat. Gas Sci. Eng..

[14] Zhou X., Ran Q. (2023). Optimization of fracturing parameters by modified genetic algorithm in shale gas reservoir. Energies.

[15] Al-Mudhafar J.W. (2019). Polynomial and nonparametric regressions for efficient predictive proxy metamodeling: application through the CO2-EOR in shale oil reservoirs. J. Nat. Gas Sci. Eng..

[16] Piyush P., Steve G., Richard M.D. (2018).

[17] Zhao X., Shen W., Wang G. (2021). Early prediction of sepsis based on machine learning algorithm. Comput. Intell. Neurosci..

[18] He Z., Fu J., Xi S. (2003). Geological features of reservoir formation of Sulige gas field. Acta Pet. Sin..

[19] Yang H., Fu J., Liu X. (2012). Formation conditions and exploration technology of large-scale tight sandstone gas reservoir in Sulige. Acta Pet. Sin..

[20] He G., Li J., Wang J. (2011). New progress and outlook of development technologies in the Sulige Gas Field. Nat. Gas. Ind..

[21] Lu C., Zhang S., Xue D. (2022). Improved estimation of coalbed methane content using the revised estimate of depth and CatBoost algorithm: a case study from southern Sichuan Basin, China. Comput. Geosci..

[22] Khan A.M., BinZiad A., Subaii A.A. (2021). Abu Dhabi Int. Pet. Exhib. & Conf.

[23] Yakoot M.S., Ragab A.M.S., Mahmoud O. (2021). SPE Annual Technical Conference and Exhibition.

[24] Marquez F.J. (2021). Offshore Technology Conference.

[25] Rathnayake S., Rajora A., Firouzi M. (2022). A machine learning-based predictive model for real-time monitoring of flowing bottom-hole pressure of gas wells. Fuel.

[26] Zhou F., Fan H., Liu Y. (2022). International Petroleum Technology Conference.

[27] Mousavi S.M., Jabbari H., Darab M. (2020). SPE Norway Subsurface Conference.

[28] Tang J., Fan B., Xiao L. (2021). A new ensemble machine-learning framework for searching sweet spots in shale reservoirs. SPE J..

[29] Gu Y., Zhang D., Lin Y. (2021). Data-driven lithology prediction for tight sandstone reservoirs based on new ensemble learning of conventional logs: a demonstration of a Yanchang member, Ordos Basin. J. Petrol. Sci. Eng..

[30] Khan A.M., BinZiad A., Subaii A.A. (2021). SPE/IATMI Asia Pacific Oil & Gas Conference and Exhibition.

[31] Mahdaviara M., Sharifi M., Bakhshian S. (2022). Prediction of spontaneous imbibition in porous media using deep and ensemble learning techniques. Fuel.

[32] Ikotun A.M., Ezugwu A.E., Abualigah L. (2023). K-means clustering algorithms: a comprehensive review, variants analysis, and advances in the era of big data. Inf. Sci..

[33] Petrović A., Đurišić Ž. (2021). Genetic algorithm based optimized model for the selection of wind turbine for any site-specific wind conditions. Energy.

[34] Wang L., Yao Y., Luo X. (2023). A critical review on intelligent optimization algorithms and surrogate models for conventional and unconventional reservoir production optimization. Fuel.

[35] Carpenter C. (2022). Numerical simulation of gas lift optimization uses genetic algorithm. J. Petrol. Technol..

[36] Li C., Cheng C. (2020). Abu Dhabi International Petroleum Exhibition & Conference.

